# Annotated plastome of the temperate woody vine *Muehlenbeckia australis* (G.Forst.) Meisn. (Polygonaceae)

**DOI:** 10.1080/23802359.2018.1456979

**Published:** 2018-03-27

**Authors:** Tanja M. Schuster, Moreland D. Gibbs, Michael J. Bayly

**Affiliations:** a National Herbarium of Victoria, Royal Botanic Gardens Victoria, South Yarra, Australia;; b School of BioSciences, The University of Melbourne, Parkville, Australia;; c Biomatters Ltd, Auckland, New Zealand

**Keywords:** *De novo* assembly, buckwheat family, GENEIOUS software, living collections, Southern Hemisphere, substitution rates

## Abstract

We assembled the plastome of the temperate, Southern Hemisphere liana *Muehlenbeckia australis* from high throughput sequencing data (paired-end Illumina reads) generated from total genomic DNA sequencing libraries. *M*. *australis*’ chloroplast genome sequence (GenBank: MG604297) is 163,484 bp in length and composed of long single copy (LSC; 88,166 bp) and short single copy (SSC; 13,486 bp) regions flanked by inverted repeats (IR; 30,916 bp each) typical for angiosperms. The plastome includes 131 genes comprising 83 protein-coding genes, 37 transfer RNA genes, eight ribosomal RNA genes, two possible pseudogenes, *psbL* and *rpl23* with internal stop codons, and truncated repeats of *ndhF* and *rps19* at IR boundaries.

Currently, seven species of Polygonaceae have published plastomes: four species of *Fagopyrum* Mill. (Logacheva et al. 2008; Cho et al. 2015; Wang et al. 2017) in tribe Fagopyreae, and two species of *Rheum* L. (Fan et al. 2015; Dagarova et al. 2017) and *Oxyria sinensis* Hemsl. (Luo et al. 2017) in the Rumiceae clade. These species have Northern Hemisphere distributions and an herbaceous habit. With *Muehlenbeckia australis*,**we here contribute the first Southern Hemisphere, lianaceous woody species, and member of the Polygoneae clade to the pool of available plastomes.

We extracted DNA from ca. 20 mg of silica gel dried leaf material from a specimen growing in the living collection of the Royal Botanic Gardens Victoria (RBGV, 37°49′57.6″S 144°58′54.5″E; herbarium voucher for collection number TMS13-64 deposited at MEL) using the DNeasy Plant Mini Kit (Qiagen, Chadstone, Australia). A sequencing library was generated following the protocol of Schuster et al. (in press) and sequenced with an Illumina NextSeq 500 machine at The Walter and Eliza Hall Institute of Medical Research (WEHI) using an Illumina mid-output (2 × 150 Paired End) kit. Sequencing yielded 3,356,344 reads, which were *de novo* assembled with the software GENEIOUS v R10 (Kearse et al. 2012). Steps and parameters followed Gibbs’ (2016) application note for chloroplast assembly. Annotations from *Oxyria sinensis* (NC_032031) were transferred to the final circular *de novo* plastome assembly and corrected manually, checking for start and stop codons, intron junctions, and inverted repeat (IR) boundaries.

The plastome of *M*. *australis* (GenBank: MG604297; Figure 1) is 163,484 bp long and has the typical four-part structure of angiosperms including two IR regions, each 30,916 bp in length, connecting the long single copy (LSC) region spanning 88,166 bp and a short single copy (SSC) region of 13,486 bp. In *M*. *australis*, the junction between LSC and IR_B_ (*J*_LB_) lies within the *rps19* gene, which is 279 bp long. Therefore, IR_A_ includes 108 bp of a truncated inverted repeat of *rps19* at *J*_LA_. The *J*_SA_ junction between SSC and IR_A_ lies in the *ndhF* gene, which is 2244 bp long, and therefore, IR_B_ includes 62 bp of a truncated inverted repeat of *ndhF* at *J*_SB_. There are two possible pseudogenes, *rpl23* and *psbL*, which have internal stop codons. However, Logacheva et al. (2008) note that RNA may be edited to produce protein-coding copies, which could rescue functionality of these presumably essential genes involved in protein synthesis and photosynthesis.

**Figure 1.**
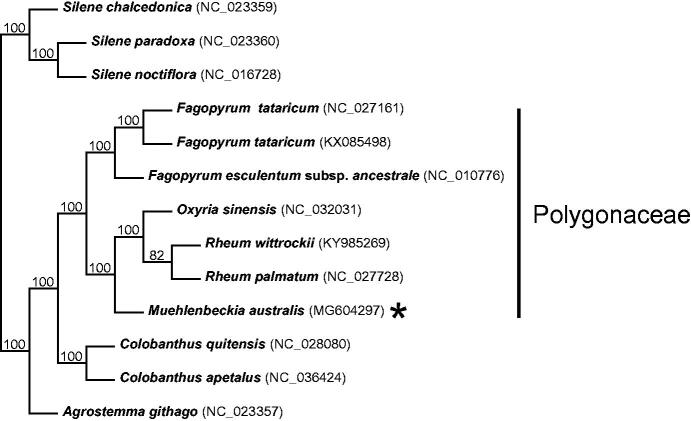
Cladogram resulting from a Neighbour-Joining (NJ) analysis including all currently available Polygonaceae plastomes and outgroup species (remainder of species not included in Polygonaceae clade indicated by a bar to the right of the tree). The alignment, NJ analysis, and tree rendering (Robinson et al. 2016) were generated on the MAFFT online server (Katoh et al. 2017), using alignment strategy FFT-NS-2 and leaving all other parameters at default settings. Bootstrap resampling = 100 with values shown on branches. *Muehlenbeckia australis* (MG604297) is indicated with an asterisk.

Sequencing of additional woody species of Polygonaceae will facilitate studies of shifts in substitution rates comparing herbaceous and woody species.
